# Osteoporosis as the First Sign of Cushing’s Disease in a Thin 16-Year-Old Boy—A Case Report

**DOI:** 10.3390/jcm12185967

**Published:** 2023-09-14

**Authors:** Anna Łupińska, Sara Aszkiełowicz, Grzegorz Zieliński, Renata Stawerska, Andrzej Lewiński

**Affiliations:** 1 Department of Paediatric Endocrinology, Medical University of Lodz, 93-338 Lodz, Poland; anna.lupinska@umed.lodz.pl (A.Ł.); renata.stawerska@umed.lodz.pl (R.S.); 2 Department of Endocrinology and Metabolic Diseases, Polish Mother’s Memorial Hospital—Research Institute, 93-338 Lodz, Poland; aszkielowiczsara@gmail.com; 3 Department of Neurosurgery, Military Institute of Medicine—National Research Institute, 04-141 Warsaw, Poland; gzielinski@wim.mil.pl; 4 Department of Endocrinology and Metabolic Diseases, Medical University of Lodz, 93-338 Lodz, Poland

**Keywords:** Cushing’s disease, obesity, growth retardation, osteoporosis

## Abstract

Cushing’s disease (CD) is an extremely rare diagnosis in children. In this report, we present the case of an almost 16-year-old, short and thin boy with CD, the first symptoms of which were spinal pain and vertebral fractures as a result of osteoporosis. In light of his growth retardation and short stature, the boy underwent diagnostics, which excluded growth hormone (GH) deficiency, hypothyroidism and celiac disease. Finally, based on cortisol profile results, dexamethasone suppression tests and bilateral sampling during catheterization of the inferior petrosal sinuses, CD was diagnosed.

## 1. Introduction

Cushing’s disease (CD) is an extremely rare diagnosis in children; however, if it occurs, it is more likely to present in older children [1,2]. It is a type of ACTH-dependent Cushing’s syndrome (CS), in which the pituitary gland is the source of ACTH secretion. The highest incidence of CD occurs in children aged 12.3–14.1 years [3]. The incidence of CD during this developmental age is approximately 5% of that seen in adults (with an annual incidence of 0.89–1 per million pediatric patients) [1,2,4]. The rarest form of ACTH-dependent CS in children is ectopic Cushing’s syndrome (ECS), associated with ectopic production of ACTH or CRH, most commonly by neuroendocrine tumors such as bronchial carcinoids, gastrointestinal tumors, medullary thyroid carcinoma, or pheochromocytomas [2,4,5]. Children with ECS constitute 1% of patients with CS in the developmental age [2]. An even rarer disease is ACTH-independent Cushing’s syndrome—associated with adrenal lesions (adenoma, carcinoma, bilateral macronodular adrenal hyperplasia (BMAH), or primary pigmented nodular adrenocortical disease (PPNAD)) [2].

Regarding CD, ACTH is secreted in an overwhelming majority of cases by pituitary corticotropic microadenomas and—less commonly—by macroadenomas, the latter occurring in only 10% of adult CD cases and even more rarely in children (2%) [1,3]. Long-term hypercortisolemia can also lead to bone-mineralization disorders, including osteoporosis, especially in the bones of the central skeleton [4,6,7].

In children, the most common features of CD are rapid weight gain (93–98%), growth retardation (63–100%) and/or facial changes (63–100%) [4]. Mood disturbances, muscle weakness, osteopenia, and headaches are less frequent symptoms. Limited data are available about bone mineral density (BMD) in children with CD. Lonser et al. [8] observed fractures in 7% of patients with CD that were studied. Chronic glucocorticoid excess associated with CD has negative effects on bone turnover, leading to bone-mineralization disorders in both adults and children. Multiple factors contribute to decreased bone mineral density in CD, including the direct effect of glucocorticoids on osteoclasts and osteoblasts, both impairing bone formation and enhancing bone resorption. Glucocorticoids also act to decrease gastrointestinal calcium absorption and renal calcium reabsorption. Bone loss occurs more frequently in Cushing’s syndrome caused by adrenal tumors in CD [6,7].

An additional factor involved in bone-mineralization disorders, particularly in adult patients with CD, may be hypogonadotropic hypogonadism. Reproductive and sexual dysfunctions are highly prevalent in CS, with higher frequency observed in patients with pituitary-related CS, compared to those with adrenal-related CS. Hypogonadism is identified in as much as 50–75% of men with CS and menstrual irregularities are present in 43–80% of women diagnosed with this condition. During active disease, there is a significant reduction in plasma testosterone and gonadotropin levels in men [7,9]. These testosterone levels typically normalize during remission of the disease. Pivonello et al. [7] suggest that the lack of testosterone normalization three months after CS treatment indicates the need for administration of testosterone to protect the patient’s bone mass. In children, cortisol excess can also suppress gonadotropin, TSH and growth hormone secretion, contributing to the absence of pubertal characteristics or inhibiting its progression in patients who have already entered puberty [1].

So far, to our knowledge, there have been no reports on children where bone-mineralization disorders (without weight gain and hirsutism) are the first sign of CD.

## 2. Case Presentation

We present the case of an almost 16-year-old boy with short stature who, in May 2021, was referred to the Osteoporosis Outpatient Clinic of the Polish Mother’s Memorial Hospital—Research Institute (PMMH-RI) in Lodz, Poland, due to severe back pain. Low bone mass was diagnosed via dual-energy X-ray absorptiometry (DXA).

Initially, it seemed that the occurrence of those symptoms might be related to steroid therapy, because in November 2020 (just after SARS-CoV-2 infection) the child had developed severe abdominal pain, accompanied by an increase in the activity of liver enzymes, and after excluding an infectious cause, autoimmune hepatitis was diagnosed. Deflazacort (Calcort) therapy was prescribed in gradually reduced doses, with the initial dose being 24 mg in the morning and 18 mg in the afternoon. This therapy was discontinued on 1 October 2021. As early as on the fifth day of glucocorticosteroid treatment, pain presented in the lumbar spine region, increasing with movement. Initially, the pain was intermittent, then it became constant. No painkillers were needed. On 7 May 2021, on the basis of DXA, low bone mass was diagnosed (Z-score Spine: −4.2, Z-score TBLH: −1.9). In June 2021 (while still undergoing treatment with steroids) the boy was admitted to the Department of Endocrinology and Metabolic Diseases PMMH-RI for further diagnostics (Table 1).

The patient was a second child, born at 40 weeks of gestational age, weighing 4150 g, measuring 56 cm, and achieving a 10-point Apgar score. During infancy, he received vitamin D supplementation in accordance with Polish recommendations at that time. However, after his first year of life, the supplementation was not taken regularly. The boy received vaccinations according to the standard immunization schedule. There was no significant family medical history.

**Figure 1:**
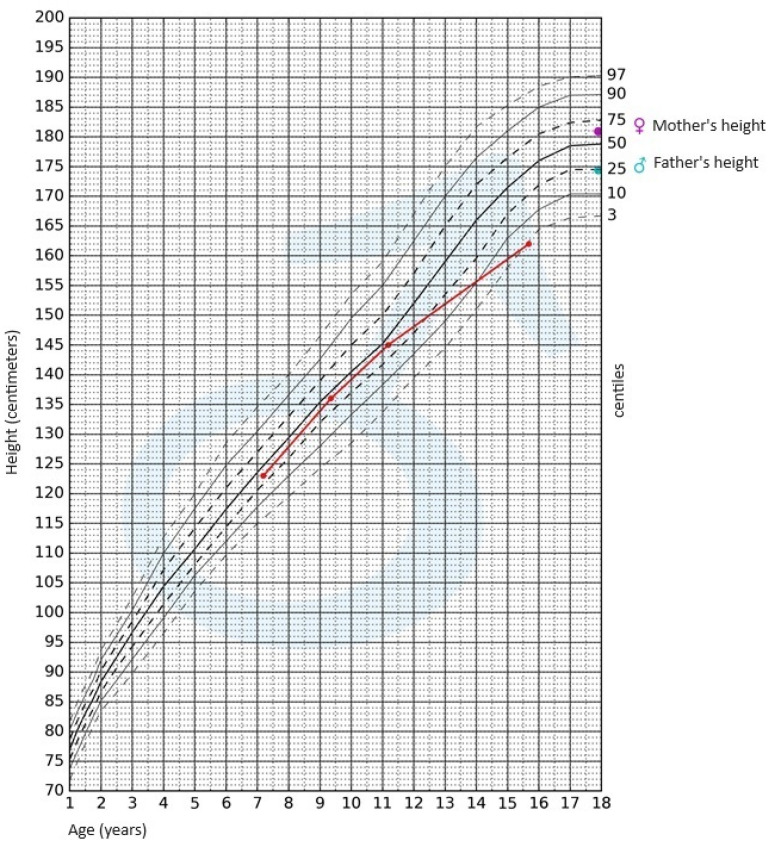
Growth chart for boys. The red line represents growth retardation from the age of 11.

During the physical examination, apart from the presence of short stature, no other notable abnormalities were detected. The skin was clear, without pathological lesions; no features of hyperandrogenism were observed. The boy’s body weight was 47.4 kg (3rd–10th centile); his height, 162 cm (<3rd centile); and height SDS, −2.36; while his BMI was 18.06 kg/m^2^ (10th–25th centile). Pubarche was assessed as stage 4 according to the Tanner scale; the volume of the testes was 10–12 mL each. After available anthropometric measurements from the patient’s medical history were plotted on the growth chart for sex and chronological age, it became evident that the boy experienced growth retardation from the age of 11 (Figure 1).

**Figure 2:**
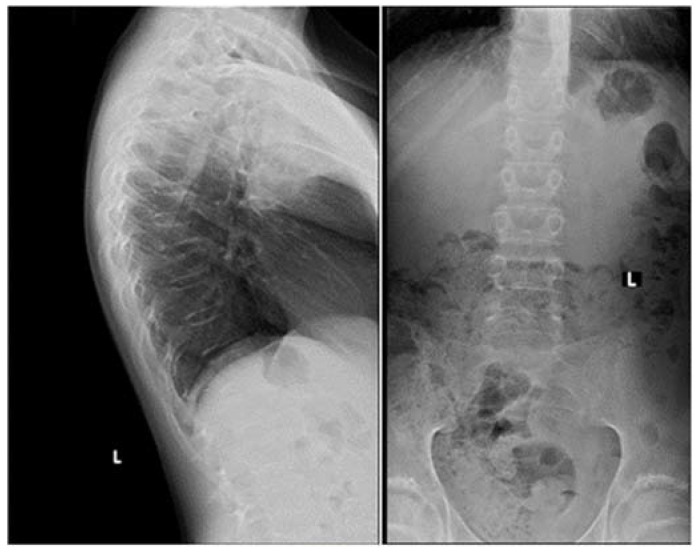
A thoracolumbar spine X-ray with multilevel vertebral fractures. Decrease in the height of the Th5–Th9 vertebrae and central lowering of the upper border plate of the L4 and L5 were found.

Apart from slightly increased calcium excretion in the 24 h urine collection (Calcium: 9.52 mmol/24 h), there were no significant abnormalities in the laboratory tests assessing calcium–phosphate metabolism (Calcium: 2.41 mmol/L, Phosphorus: 1.3 mmol/L). Serum parathormone (PTH) and vitamin D concentrations remained normal (PTH: 22.9 pg/mL, 25(OH)D: 46.7 ng/mL). Due to the described pain complaints, a thoracolumbar spine X-ray was performed. A decrease in the height of the Th5-Th9 vertebrae and central lowering of the upper border plate of the L4 and L5 were observed (Figure 2).

Magnetic resonance imaging (MRI) of the spine confirmed multilevel vertebral fractures, which, together with the presence of low bone mass on DXA examination, allowed a diagnosis (according to ISCD guidelines) of osteoporosis to be made. Treatment included calcium supplements and cholecalciferol. The parents did not consent to treatment with bisphosphonates (sodium pamidronate), which is an off-label treatment.

In light of the patient’s short stature and growth retardation, an endocrinological assessment was conducted. The possibility of growth hormone (GH) deficiency and hypothyroidism as underlying causes for the growth retardation was ruled out. Gonadotropin and androgen levels were adequate for the pubertal stage (FSH—8.3 IU/L, LH—4.7 IU/L, testosterone—4.750 ng/mL, DHEA-S—230.30 µg/dL (normal range: 70.2–492), 17-OH-progesterone—0.78 ng/mL). The bone age was assessed to be 15 years.

**Figure 3:**
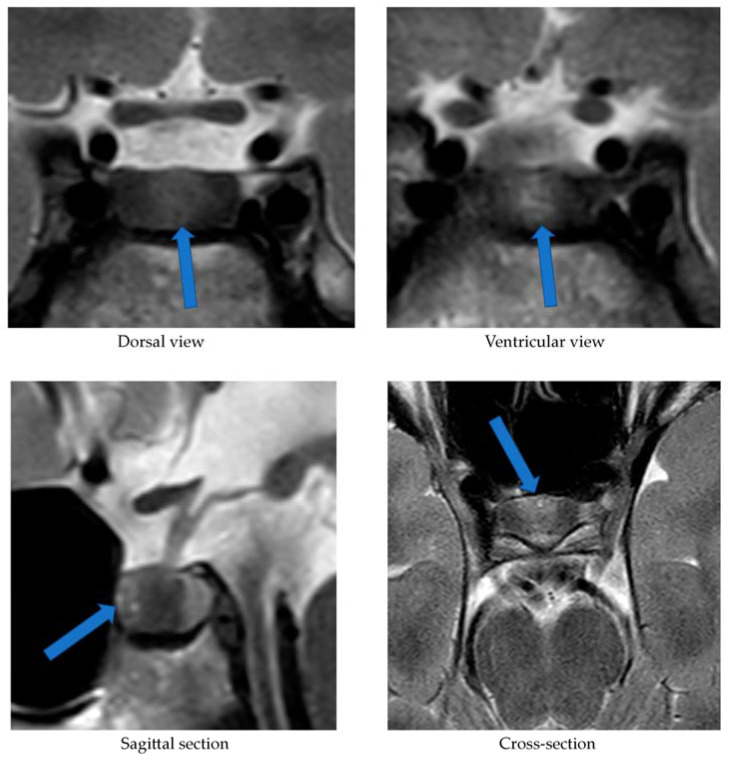
MRI examination image depicting the poorly demarcated area in the anterior part of the glandular lobe. The arrows point to a structure suspected of being an adenoma.

Alongside continued steroid therapy for autoimmune hepatitis, profiles of cortisol and ACTH secretion were performed. Due to the patient’s elevated cortisol levels during night hours (cortisol 24:00—10.7 µg/dL), an overnight dexamethasone suppression test (DST) and low-dose dexamethasone suppression test (LDDST) were performed. After administering 1 mg dexamethasone (23:00), his morning cortisol level (8:00) still remained elevated (cortisol—3.4 µg/dL). However, after administering 0.5 mg dexamethasone every 6 h for the next 2 days, cortisol levels (8:00) normalized (cortisol—1.0 µg/dL). An MRI of the pituitary gland showed only a poorly demarcated area in the anterior part of the glandular lobe, measuring approximately 2.0 × 3.5 × 5.0 mm on T2W images (Figure 3). A follow-up MRI examination was recommended, which was performed during the child’s next hospitalization in January 2022. The previously described area was still very faint.

**Figure 4:**
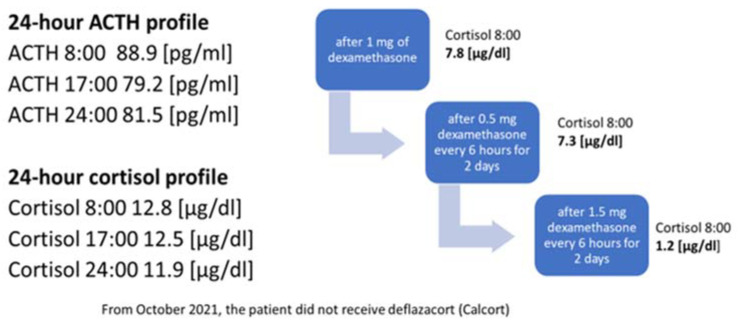
Laboratory findings indicating the diagnosis of ACTH-dependent Cushing’s syndrome.

In October 2021, the administration of deflazocort as a treatment was discontinued. During hospitalization in January 2022, the diurnal pattern of ACTH and cortisol secretion was re-evaluated, yet no consistent diurnal rhythm was observed; cortisol levels remained elevated at night. For this reason, overnight DST and then LDDST were carried out again (Figure 4), in which no suppression of cortisol concentrations was obtained. Only after a high-dose DST (HDDST), in which a high 1.5 mg of dexamethasone was administered every 6 h (125 µg/kg/24 h), was cortisol secretion suppressed.

Based on the above results, CD was suspected as the cause of osteoporosis and growth retardation. In February 2022, a CRH test was performed upon the patient, which revealed a four-fold increase in ACTH levels and a two-fold increase in serum cortisol levels (Table 2).

The CRH stimulation test was administered in the morning using human synthetic CRH (Ferring) at a dose of 1 μg/kg of body weight. During the test, cortisol and ACTH levels were measured in serum at the following time points: −15, 0, 15, 30, 60, and 90 min (see Table 2). As part of the diagnostic process, urinary free cortisol excretion was also measured over two consecutive days. Only on the first day was there a slight elevation in urinary free cortisol concentration, measuring 183.60 μg/24 h (normal range: 4.3–176). The measurement performed on the second day showed a normal urinary free cortisol concentration of 145.60 μg/24 h (normal range: 4.3–176). On 2 March 2022, the patient underwent a bilateral inferior petrosal sinus sampling (BIPSS). Human CRH stimulation was also used during the procedure. The presence of ACTH-dependent hypercortisolemia of pituitary origin was confirmed. The outcome of the CRH stimulation during the BIPSS is presented in Table 2. The boy qualified for transsphenoidal surgery (TSS) of the pituitary adenoma and was successfully operated on (8 March 2022). Postoperative histopathological examination revealed features of a corticotroph-rich pituitary adenoma.

## 3. Discussion

Osteoporosis, like CD, is extremely rare in the developmental age population. Bone-mineralization disorders among children may be primary (e.g., osteogenesis imperfecta), or secondary to other diseases or their treatment (e.g., with glucocorticosteroids). This case report presents a boy with osteoporosis, the cause of which was originally attributed to the treatment of autoimmune hepatitis with glucocorticosteroids. Steroid therapy is the most common cause of bone-mineralization disorders in children. However, osteoporosis is a late complication of steroid treatment. Briot et al. [10] demonstrated that the risk of fractures increases as early as 3 months after initiating steroid therapy. An additional factor increasing the risk of fractures is the dose of glucocorticosteroids used, corresponding to 2.5–5 mg of prednisolone per day [10]. In the case of the present patient, the appearance of spinal pain and thus vertebral fractures could not have been related to the deflazacort treatment started 5 days earlier. The bone-mineralization disorder must therefore have occurred much earlier. For this reason, the authors considered it necessary to search for other endocrine causes of osteoporosis development, including hypogonadism, growth hormone deficiency or Cushing’s syndrome/disease.

The serum vitamin D concentration can also influence bone mineral density. Every patient with mineralization disorders, especially with osteoporosis, requires a thorough assessment of calcium–phosphate metabolism [11]. Until the initiation of steroid therapy in March 2021, the patient did not undergo regular vitamin D supplementation. At the start of deflazacort treatment, his serum 25(OH)D concentration was 12.4 ng/mL. Consequently, additional cholecalciferol supplementation at a dose of 3000 IU/day was introduced. In a subsequent measurement conducted in June 2021, the concentration was within the reference range [25(OH)D: 46.7 ng/mL].

Considering the lack of regular supplementation before March 2021, it can be assumed that in October 2020, when the boy experienced SARS-CoV-2 infection, his serum vitamin D concentration was likely decreased as well, which could have had a further negative impact on the patient’s bone mineralization. Scientific reports indicate that adequate vitamin D levels reduce the risk of viral infections, including SARS-CoV-2 [12]. Di Filippo et al. [13] demonstrated that vitamin D deficiency observed in 68.2% of SARS-CoV-2-infected individuals correlated with a more severe course of the infection. In our patient, the course of COVID-19 was asymptomatic, and the diagnosis was established based on positive IgM antibody titers against SARS-CoV-2. The vitamin deficiency was most likely associated with irregular supplementation and lack of exposure to UV radiation (due to lockdown measures in Poland at that time). A reduced serum 25(OH)D concentration could have contributed to worsened bone mineral density and increased susceptibility to SARS-CoV-2 infection; however, it is the chronic hypercortisolism characteristic of CD that most likely led to the development of osteoporosis with accompanying fractures.

Another factor necessitating further diagnostic investigation into CD was the patient’s growth retardation observed since the age of 11. Both the pubertal state of the boy, and his gonadotropin and testosterone serum levels, allowed us to exclude hypogonadism. Maximum spontaneous nocturnal secretion of the growth hormone was 31.84 ng/mL. The diagnosis of CD was established on the basis of elevated cortisol levels at night and the lack of cortisol suppression in the test after administering dexamethasone. Final confirmation of the diagnosis was obtained in a post-CRH stimulation test. In pediatric cases, the absence of typical diurnal variation in serum corticosolemia, especially the nocturnal decline, and the inability to suppress cortisol secretion at midnight, are highly sensitive indicators of hypercortisolemia [6,8]. Consequently, in our patient, osteoporosis was a complication of diagnosed CD.

The patient in question was not obese, which is the predominant symptom of CD. This symptom, according to Ferrigno et al. [1], is present in 92–98% of examined children diagnosed with CD. Storr et al. [14] showed that facial changes and facial swelling were observed in 100% of subjects with CD, whereas Lonser et al. [8] observed this in only 63% of children with CD. In our patient, no changes in facial appearance were observed. Other symptoms typical of CS, such as hirsutism, acne, or bruises, were not noticed either. These symptoms were observed in all children with CD studied by Wędrychowicz et al. [3]. Non-specific symptoms of this condition may include mood changes, depression and emotional vacillation [1,8]. However, our patient’s parents did not observe any changes in the boy’s behavior. The indication for initiating the whole diagnostic process was (in addition to osteoporosis) growth retardation. Ferrigno et al. [1] point out that chronic hypercortisolemia most often leads to growth disorders accompanied by excessive weight gain. This is an early, highly sensitive and characteristic sign of CD. Short stature is not always observed and occurs in one in two children diagnosed with CD. The patient we present was short (height—162 cm (<3rd centile hSDS: −2.36)); growth retardation was observed from the age of 11 years.

The occurrence of vertebral fractures and the accompanying pain as the initial symptoms of hypercortisolism, the absence of obesity, and the confirmation of CD, an exceedingly rare condition in the pediatric population, collectively underscore the uniqueness of our patient’s disease presentation. A case involving a child with such an atypical course of ACTH-dependent CS has not been described before. Han et al. [15] reported a case of a 28-year-old lean woman (BMI: 19 kg/m²) with ACTH-independent CS due to a left adrenal adenoma, where, similarly to our patient, the initial manifestation of hypercortisolism was compression fractures of the thoracic vertebrae. The authors emphasize that vertebral fractures may affect 30–50% of patients with Cushing’s syndrome, with a higher frequency observed in patients with ACTH-independent CS compared to those in whom hypercortisolism results from the presence of pituitary adenoma [15].

The lack of obesity in a patient with hypercortisolism could be attributed to malnutrition, which accompanies the growth process in ECS. Hence, a crucial aspect was the differential diagnosis between CD and ECS. To this end, we performed a stimulation test using hCRH. We considered cut-off points for diagnosing CD to be a 35% increase in ACTH concentration at 15 and/or 30 min, and at least a 20% increase in cortisol concentration at 30 and 45 min [16,17]. In the case of ECS, a significant rise in CRH and cortisol concentrations is not observed. Recently published reports emphasize the need to explore new cut-off points to enhance the sensitivity and specificity of this test. Detomas et al. [5] indicate that an increase in ACTH ≥ 31% and cortisol ≥ 12% in the 30th minute of CRH tests allows for a highly sensitive and specific differentiation between CD and ECS. The authors highlight that measuring these hormones at the 60 min stage of the test does not provide diagnostic benefits. Notably, the study employed ovine CRH, which exhibits stronger and more prolonged stimulatory effects compared to the hCRH available in Europe that was used to diagnose our patient [5]. Conversely, Elenius et al. [16] suggest that optimal values for distinguishing between CD and ECS in the CRH stimulation test involve an increase in ACTH and/or cortisol levels of more than 40% during the test. In our patient, an over four-fold increase in ACTH levels and a more than two-fold increase in cortisol levels were observed at the 30 min mark of the test, thus independently and definitively excluding ECS regardless of the adopted cut-off points.

Our patient’s case also demonstrates that MRI is not a perfect method of visualizing an ACTH-secreting pituitary adenoma. In the first MRI examination performed upon our patient, a poorly demarcated area (2.0 × 3.5 × 5.0 mm) was described in the anterior part of the glandular lobe; in the examination performed 6 months later, this area maintained poor visibility, while laboratory results at the time clearly indicated an ACTH-dependent form of CS. It was only the bilateral inferior petrosal sinus sampling (BIPSS) that allowed a clear diagnosis. Data from the literature indicate that microadenomas smaller than 3–4 mm are visible on MRI in only half of cases. In two large studies including children, pituitary adenomas were found on MRI in 63% and 55% of cases [18]. Among the patients with CD studied by Wędrychowicz et al. [3], pituitary adenomas were described on MRI in all of them, but in two patients (50%) this was only achieved upon follow-up. In the standard procedure, in the absence of a pituitary lesion in the MRI examination, it is recommended that a BIPSS be performed. In the case of our patient, this examination was necessary to make a definitive diagnosis.

When analyzing the results of the BIPSS with hCRH stimulation, we employed the classical cut-offs for the ACTH IPS:P (Inferior Petrosal Sinus: Peripheral) ratio (i.e., ≥2 at baseline and ≥3 after hCRH stimulation) [1]. This allowed the confirmation of CD and determination of the pituitary adenoma’s localization, followed by the procedure for its surgical removal. The optimal cut-off values for the IPS:P ratio remain controversial. There are ongoing efforts to establish new, more precise cut-off points. Detomas et al. [19] demonstrated that an IPS:P ratio ≥ 2.1 during desmopressin stimulation in the BIPSS most accurately differentiates CD from ECS. Conversely, Chen et al. [20] showed that the optimal pre-desmopressin stimulation IPS:P ratio cut-off is 1.4, and post-stimulation it is 2.8. Both studies suggest the utilization of lower cut-off values for the IPS:P ratio than those traditionally adopted. Chen et al. [20] also advocate for avoiding stimulation during BIPSS. In most cases, the IPS:P ratio before stimulation is sufficient for diagnosing CD. According to the authors, desmopressin stimulation should be reserved for patients with ambiguous MRI findings or with a pituitary adenoma with diameter less than 6 mm. However, considering that the concentration of ACTH in the right inferior petrosal sinus in our patient was over 4 times higher than in the peripheral vessel and nearly 14 times higher after hCRH stimulation, regardless of the applied criteria, CD could be unequivocally diagnosed in our patient, and the lateralization of the microadenoma could be determined with certainty.

The rarity of CD, and the diagnostic difficulties stemming from its oligosymptomatic or atypical course, encourage description in the form of case reports. Eviz et al. [21] delineate the occurrence of cerebral cortical atrophy in two children with ECS. Additionally, other researchers have underscored the potential for thyroid disorders to manifest alongside hypercorticosolemia [22]. Although obesity typically stands out as a primary symptom of CD, Pomahacova et al. [23] reported a case involving two children with CD who maintained normal body weight, mirroring our patient’s situation. The symptoms that prompted diagnostic investigation in these instances included weakness, sleep disturbances and growth retardation. Interestingly, growth retardation, along with facial changes, was observed in all examined children with CD [23]. Nonetheless, to the best of our knowledge, we have yet to encounter a case report resembling ours. Therefore, it remains crucial to share our experiences.

## 4. Conclusions

Cushing’s disease is an extremely rare diagnosis in children. In Poland, there is no statistical record of occurrences of this disease among children. Wędrychowicz et al. reported that in their single Polish center, between 2012 and 2018, they identified four cases of children aged 7–15 who were diagnosed with CD [3]. The case we present shows that obesity, commonly considered as a predominant symptom of CD, is not necessarily observed in patients with this diagnosis in the developmental age population. Among children, it is growth disturbance that may be the first manifestation. On the other hand, a late complication of CD may be osteoporosis, so whenever a child is diagnosed with a bone-mineralization disorder, the cause of its development should be sought.

Diagnosis should be pursued until all potential causes of the described symptoms, including the rarest ones, are definitively ruled out—even if the clinical presentation, as in the case of our patient, initially does not point towards the final diagnosis. Thus far, no case of a child with CD exhibiting such subtle symptomatology has been described in the literature. The challenges in diagnosis we encountered primarily resulted from the atypical clinical outcome of CD in our patient—normal body weight, absence of hyperandrogenism, mood disturbances not apparent to caregivers and the patient’s immediate environment, as well as normal progression of puberty, did not immediately lead to the consideration of endocrinological causes of osteoporosis. The steroid therapy employed due to autoimmune hepatitis also complicated the diagnostic process. Only after discontinuing deflazacort treatment was it possible to definitively diagnose CD.

Our patient required hydrocortisone replacement in gradually decreasing doses for a year following TSS. Considering that pituitary adenomas in children can be genetically predisposed (e.g., MEN 1 mutation, AIP mutation, USP8 mutation, and other rarer ones), genetic consultation was sought [1]. However, the conducted tests have thus far excluded the most common mutations in our patient. Due to the diagnosed osteoporosis, chronic supplementation with calcium and cholecalciferol was recommended, along with annual follow-up DXA scans. Studies indicate that patients in remission from CD experience a gradual improvement in bone mineral density [3]. While we can currently observe remission in our patient’s case, the advanced bone age of the child (indicating the completion of the growth process) left limited potential for significant improvement in final growth. The patient still requires regular endocrinological and neurosurgical follow-ups, hormonal assessments, and pituitary MRI examinations.

## Figures and Tables

**Table 1:** The medical history and the course of diagnostics and treatment.

	Date	Calendar of Medical Events and Diagnostic/Therapeutic Procedures
	October 2020	SARS-CoV-2 infection
	November 2020	Abdominal pain with elevation of liver enzymes
← Steroid treatment →	March 2021	Autoimmune hepatitis was diagnosed Start of steroid treatment
	March 2021 (5 days after beginning of steroid treatment)	Onset of back pain
	May 2021	DXA—low bone mass
	June 2021	First hospitalization, X-ray of spine, diagnosis of osteoporosis, growth retardation was noticed, the onset of diagnostic process—overnight DST (no suppression) LDDST (suppression)
	August 2021	MRI of pituitary gland
	October 2021	Discontinuation of steroid treatment
	January 2022	Control MRI of pituitary gland and overnight DST (no suppression) LDDST (no suppression), HDDST (suppression)
	February 2022	CRH stimulation test
	2 March 2022	Bilateral inferior petrosal sinus sampling (BIPSS)
	8 March 2022	Transsphenoidal surgery (TSS)
	28 March 2022	Histopathological examination

**Table 2:** The results of human CRH (hCRH) stimulation test and bilateral inferior petrosal sinus sampling (BIPSS).

Human CRH Stimulation Test’s Results				
Time Point	ACTH [pg/mL]	%	Cortisol [µg/dL]	%
−15 min	67		16.2	
0 min	67		15.4	
15 min	354.5	529	24.2	157
30 min	276	412	32.7	212
60 min	148	221	35.2	145
90 min	82	122	28.0	116
	ACTH post-CRH peak level [pg/mL]: 354		Cortisol post-CRH peak level [µg/dL]: 35.1	
**Bilateral Inferior Petrosal Sinus Sampling (BIPSS)**				
**Time Point**		**ACTH [pg/mL]**		
		**From Peripheral Vein (P)**	**From Left Inferior Petrosal Sinus (IPS)**	**From Right Inferior Petrosal Sinus (IPS)**
−1 min		56.1	65.6	301.0
0 min		69.6	58.0	285.9
1 min		62.9	67.4	694.6
3 min		88.3	119.5	1231.0
5 min		98.3	117.4	1054.0
10 min		104.6	118.9	636.1
15 min		98.9	110.4	358.5
Basal ACTH IPS/P ratio		4.1:1		
Post-hCRH ACTH IPS/P ratio		13.9:1		

## Data Availability

Not applicable.
